# Accident vasculaire cérébral ischémique, complication inhabituelle d’une morsure de vipère: à propos d’un cas

**DOI:** 10.11604/pamj.2022.41.50.22225

**Published:** 2022-01-18

**Authors:** Mouhssine Assamadi, Yassine Ait M´Barek, Yassine Elallouchi, Lamia Benantar, Khalid Aniba

**Affiliations:** 1 Service de Neurochirurgie, Hôpital Ibn Tofail, CHU Mohammed VI Marrakech, Faculté de Médecine et de Pharmacie de Marrakech, Université Cadi Ayyad, Marrakech, Maroc

**Keywords:** Morsure de vipère, cérastes, AVC ischémiques, cas clinique, Snake bite, cerastes, ischemic stroke, case report

## Abstract

La morsure de serpent constitue la forme la plus grave des envenimations. Son incidence annuelle dépasse six millions avec une mortalité non négligeable. Les morsures par vipère sont souvent responsables d´une coagulopathie à l´origine d´un syndrome hémorragique, cependant les complications ischémiques sont rares. Nous rapportons l´observation d´une fille de 6 ans victime d´une morsure de vipère identifiée comme vipère cérastes au niveau de la cheville gauche, admise 4 jours après la morsure en trouble de conscience avec un tableau de coagulation intra-vasculaire disséminée. L´examen a mis en évidence une hémiparésie gauche et un syndrome vipérin au membre inferieur gauche. La tomodensitométrie cérébrale a objectivé une ischémie temporo-pariétale droite. L´évolution a été marquée par l´aggravation neurologique nécessitant une ventilation mécanique. Les accidents vasculaires cérébraux, en particulier ischémiques secondaires à une envenimation vipérine grave sont exceptionnels. Le mécanisme physiopathologique n´est pas clairement élucidé mais semble être multifactoriel nécessitant plusieurs recherches.

## Abstract (en)

Snake bite is the most severe type of envenomation. Annual incidence exceeds six million, with high mortality rates. Viper bites are often responsible for coagulopathy, which causes bleeding disorders. However, ischemic complications are rare. We here report the case of a 6-year-old girl victim of a viper [genus Cerastes] bite in the left ankle. She was admitted with disorders of consciousness and disseminated intravascular coagulation 4 days after the bite. Clinical examination showed left hemiparesis and left lower limb viperin syndrome. Brain CT scan objectified right temporoparietal ischemia. The patient outcome was marked by neurological worsening, requiring mechanical ventilation. Stroke, in particular ischemic stroke secondary to severe snake envenomation, is exceptional. The physiopathological mechanism has not been clearly elucidated but it appears to be multifactorial. Then new studies are needed.

## Introduction

La morsure de serpent (MS) constitue la forme la plus grave des envenimations. L´incidence annuelle des MS dépasse six millions dans le monde [1]. Au Maroc en 2017, le centre antipoison et de pharmacovigilance a recensé 408 déclarations de MS avec 8 cas de décès soit une létalité de 1,96% [2]. Les accidents vasculaires cérébraux (AVC) secondaires à une envenimation vipérine grave sont exceptionnels, surtout pour les AVC ischémiques. Le mécanisme physiopathologique n´est pas clairement élucidé, mais semble multifactoriel [3]. Nous rapportons le cas d´un AVC ischémique suite à une morsure de vipère à cornes de l´espèce cérastes cérastes.

## Patient et observation

**Informations relatives aux patients:** fille de 6 ans, avec un carnet de vaccination à jour, sans antécédents pathologiques particuliers. Admise aux urgences 4 jours après une morsure de vipère identifiée comme Cerastes cerastes au niveau de la cheville gauche.

**Figure 1:**
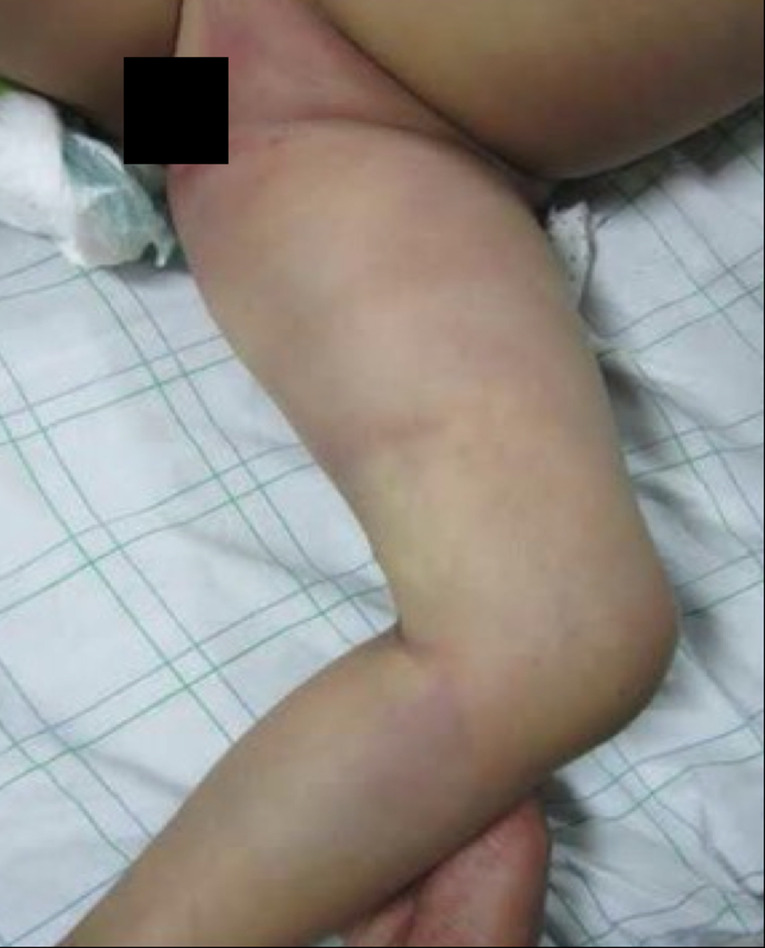
œdème et ecchymose extensive au niveau du membre inférieur gauche, étendu jusqu'à la racine de la cuisse chez notre patiente

**Résultats cliniques:** l´examen clinique à l´admission a objectivé une patiente confuse avec un score de Glasgow (GCS) à 14/15, des conjonctives décolorées, une tension artérielle à 70/40 mmHg, une fréquence cardiaque à 130 battements/min et une fréquence respiratoire à 26 cycles/min. L´examen neurologique a trouvé une hémiparésie gauche proportionnelle (testing musculaire à 4/5). L´examen du membre inferieur gauche a mis en évidence des traces de crochets au niveau de la cheville associés à un œdème et des ecchymoses d´extension rapide arrivant jusqu´à la racine de la cuisse (Figure 1), avec une douleur à la mobilisation du membre et un saignement modéré au niveau du site de la morsure. Les pouls pédieux et tibial étaient bien perçus.

**Figure 2:**
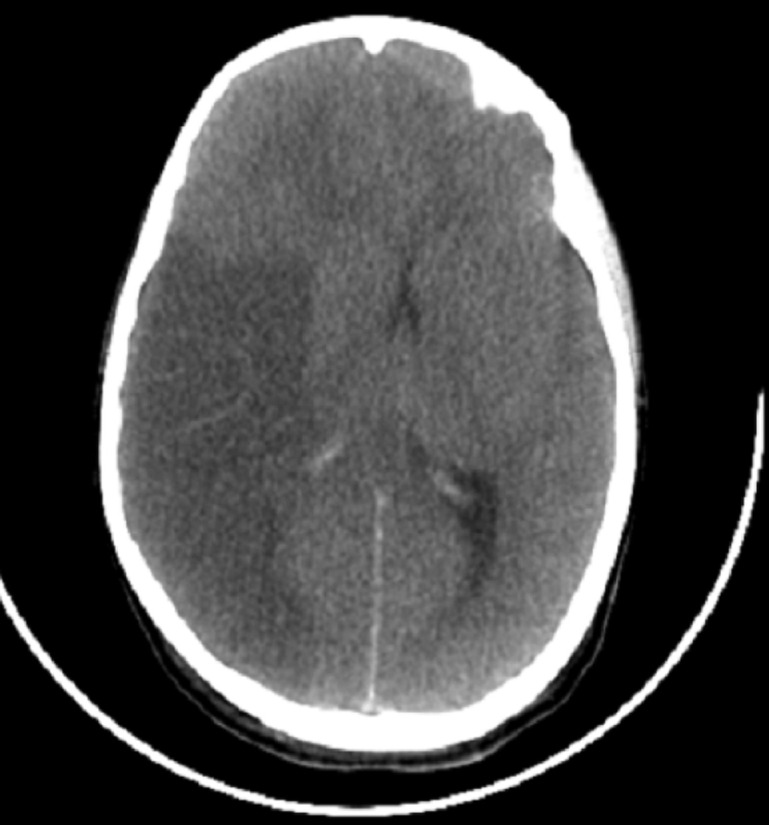
foyer d´ischémie systématisé temporo-pariétal droit avec une hémorragie méningée et un effet de masse sur le ventricule latéral homolatéral

**Démarche diagnostique:** à l´admission, une TDM cérébrale a été faite qui a objectivé un foyer d´ischémie systématisé temporo-pariétal droit avec une hémorragie méningée et un effet de masse sur le ventricule latéral homolatéral et un début d´engagement sous-falcoriel (Figure 2). A J2 d´hospitalisation (J5 de la morsure), il y a eu une aggravation de l´état neurologique (GCS à 7/15 vs 14/15 à l´admission), les pupilles étaient isoréactives. Le bilan biologique a mis en évidence une anémie à 5 g/L normochrome normocrocytaire, une leucopénie à 1200 éléments/mm, une thrombopénie à 35000 éléments/mm^3^, un taux de prothrombine à 35%, urée à 0,35 g/L, fibrinogène à 0,4 g/L, et une créatine phosphokinase (CPK) à 2364 UI/l (plus de 20 fois la normale). La radiographie du thorax et l´électrocardiogramme étaient sans anomalies. Devant cette aggravation neurologique et l´aspect scanographiques, la mise en place d´un dispositif intraparenchymateux pour mesurer la pression intra crânienne (PIC) a été jugé primordiale pour guider notre attitude thérapeutique. Malheureusement à défaut de matériel nécessaire pour la mesure invasive de PIC; nous n´avons pas pu effectuer cette intervention.

**Intervention thérapeutique:** un traitement initial en urgence a été administré à base d´une oxygénothérapie, un remplissage vasculaire par des cristalloïdes, une antibiothérapie probabiliste par amoxicilline-acide clavulanique et une analgésie par des morphiniques. Le traitement spécifique s´est basé l´administration du sérum antivénérien FAV-Afrique®. Après l´aggravation (J2 d´hospitalisation) la patiente a été intubée-ventilée-sédatée, et un traitement médical de l´hypertension Intra crânienne (HTIC) a été instauré. Une craniotomie décompressive a été discutée mais jugée risquée en raison des désordres biologiques et en l´absence de mesure de la valeur de la PIC permettant d´affirmer avec certitude la présence d´une HTIC.

**Suivi et résultats:** malgré la correction des troubles biologiques, l´évolution de l´état neurologique de la patiente a été défavorable et le décès est survenue à J7 de la morsure.

## Discussion

Au Maroc la fréquence des envenimations de vipère reste sous-estimée du fait de la sous-notification globale des intoxications par les médecins des différentes provinces. Les deux familles de serpents venimeux les plus rencontrées sont les élapidés représentés par le cobra ou le naja-haje, et les Viperidae comprenant sept espèces (*Bitis arientans, Cerastes cérastes, Cerastes vipera, Vipera latastei, Daboia mauritanica, Vipera monticala, Echis carinatus*) [4].

Contrairement au syndrome cobraïque, l´injection du venin de vipère est rapidement suivie d´un désordre de l´hémostase comprenant des troubles locaux (œdème, douleur, nécrose voire gangrène gazeuse) et un syndrome hémorragique [3]. Les troubles neurologiques sont exceptionnels. Ils peuvent être secondaires soit à une défaillance circulatoire suite à une hypotension aigue par vasodilatation artérielle et/ou hématologique suite à un vasospasme dû à un saignement périvasculaire, soit à une action directe du venin neurotoxique de certaines populations de vipères. Ce dernier, est responsable quelques heures après la morsure d´une atteinte neurologique périphérique. Il s´agit le plus souvent d´un ptosis, mais d´autres signes d´atteinte des nerfs crâniens sont rapportés: ophtalmoplégie, diplopie, dysarthrie, agueusie, paralysie de l´orbiculaire des lèvres, troubles de la déglutition et de l´accommodation. Un syndrome neurologique plus complet peut apparaître, avec somnolence, vertiges, dyspnée et paresthésies diffuses [3,5]. L´atteinte neurovasculaire après une morsure de vipère est rare, c´est surtout l´apanage des envenimations par certains crotales sud-américains du genre Bothrops: Bothrops lanceolatus et Bothrops caribbaeus [6]. Il peut s´agir d´un accident vasculaire cérébral (AVC) hémorragique, et exceptionnellement d´un infarctus cérébral. Dans une étude en Equateur, dans une série de 309 patients, seules huit complications vasculaires cérébrales (2,6%) ont été signalées, dont sept de nature hémorragique et une seule de type ischémique [7]. Chani *et al*.rapportent un cas d´AVC ischémiques multiples suite à une morsure grave de vipère de l´espèce Cerastes cerastes [3]. Chez notre patiente la vipère responsable de la morsure était une vipère Cerastes cerastes, identifiée par des témoins oculaires, occasionnant une envenimation grave, avec des signes locaux et des signes systémiques dominés par l´atteinte neurologique, le collapsus vasculaire et la coagulation intravasculaire disséminée (CIVD).

Chaque venin possède des enzymes favorisants ou inhibants la coagulation à plusieurs niveaux. Le venin de la vipère *Cerastes cerastes* possède de nombreuses protéines impliquées dans les AVC ischémiques telles que: la proteinase RP 34, l´afaacytine, la protéine proagrégante de *Cerastes cerastes*, la cerastocytine, la cerastotine, la cerastobine et la viperabine. L´ensemble peut entrainer l´activation pathologique de l´hémostase par adhésion et agrégation plaquettaire suite à l´activité des sérines protéases, et/ou par l´activation de la prothrombine ou des facteurs V et X suite à l´action d´enzymes « thrombine-like» [3,6,8,9]. Cependant, ces phénomènes vont aboutir à la formation de microthrombi qui peuvent provoquer des infarcissements viscéraux à distance, auxquels ont été attribués les accidents vasculaires cérébraux [3], les infarctus du myocarde, les embolies pulmonaires [6], les thromboses de l´artère fémorale, la nécrose extensive des parties molles.

La multiplicité des enzymes d´un même venin explique la difficulté d´éviter un syndrome hémorragique ou thrombotique en agissant sur une seule étape de l´hémostase. L´immunothérapie antivenimeuse, qui agit globalement sur les différents constituants du venin, demeure l´unique thérapeutique spécifique de l´envenimation ophidienne [10]. Le traitement des complications neurovasculaires est essentiellement préventif, car une fois installées, leur pronostic est souvent défavorable. Il nécessite l´administration d´un antivenin adapté, si possible dans les six premières heures suivant la morsure [10]. Alors que dans notre cas, la patiente a reçu l´antivenin après 4 jours de la morsure, à cause du retard d´acheminent de la patiente dans notre structure. L´héparinothérapie, qui aggrave les troubles engendrés par les hémorragines, les désintégrines, voire les enzymes fibrinolytiques, est contre-indiquée, et son inefficacité a été démontrée par des études randomisées [10]. Son introduction à titre préventif n´est justifiée qu´à distance de la morsure, une fois que les paramètres biologiques de l´hémostase sont corrigés [6].

**Point de vue des parents de la patiente:** ils étaient conscients du pronostic, et la gravité que l´état de leur fille.

**Consentement éclairé:** il a été obtenu auprès des parents de la patiente pour que nous puissions utiliser ses photos ainsi que les informations cliniques et images radiologiques pour ce rapport de cas.

## Conclusion

L´envenimation par morsure de vipère est un accident grave, la venimosité des espèces à des conséquences néfastes sur la morbidité et mortalité des victimes. Les atteintes cérébrales et en particulier ischémiques sont rares mais graves nécessitant plusieurs recherches afin de mieux connaître les mécanismes physiopathologiques et d´adapter les stratégies thérapeutiques.
